# Anaesthetic challenges in a child with perforated appendicitis and COVID-19 Co-Infection: A case report

**DOI:** 10.1016/j.amsu.2021.102931

**Published:** 2021-10-12

**Authors:** Annie Maxwell, Kai Ming Teah, May Zaw Soe, Boon Tat Yeap

**Affiliations:** a Department of Anaesthesiology and Intensive Care, Hospital Queen Elizabeth, 88350, Kota Kinabalu, Sabah, Malaysia; b Department of Anaesthesiology and Intensive Care, Faculty of Medicine and Health Sciences, Universiti Malaysia Sabah, 88400, Kota Kinabalu, Sabah, Malaysia; c Department of Medical Education, Faculty of Medicine and Health Sciences, Universiti Malaysia Sabah, 88400, Kota Kinabalu, Sabah, Malaysia

**Keywords:** *COVID-19*, *Child*, *Perforated appendicitis*, *Appendicectomy*, *Personal protective equipment*, *Problem*

## Abstract

**Background:**

The deadly coronavirus disease 2019 (COVID-19) wreaked havoc globally in early 2020 and caused lives to a standstill. Healthcare workers (HCW) handling patients infected with COVID-19 wore protective equipment to defend themselves from cross infection and curbing further spread. Nevertheless, these do hamper their dexterity, especially during surgical procedures.

**Case presentation:**

A child presented to our centre needing an emergency open appendicectomy during the coronavirus disease 2019 (COVID-19) pandemic in June 2021. Prior to the surgery, her initial test for COVID-19 was negative but subsequently became positive on the second test. Fortunately, all HCW during the care for the patient, donned full personal protective equipment (PPE), and avoided cross-infection.

**Discussion:**

HCW handling patients with COVID-19 should wear adequate PPE to. However, these pose detrimental effects to their dexterity during routine care of such patients. Good teamwork and communication among HCW and parents are important during the safe management of a young child with COVID-19.

**Conclusion:**

HCW should have low index of suspicion of COVID-19 in children with upper respiratory tract infection. Prompt and pro-active measures should be rapidly taken to prevent exposure and co-infection. Wearing multi layers of PPE do negatively affect the mood and agility of HCW handling young children with COVID-19. Thus, they should practice good team work, receive regular simulation and scenario-based training to be better prepared for pressurised situations.

## Highlights

• Challenges faced during management of a child in a resource limited nation during COVID-19 pandemic.
• Healthcare workers are at risks of contracting COVID-19 infections from patients.
• Various methods were implemented to curb the spread of COVID-19 in the operating theatre and hospital.

## 1 Introduction

The COVID-19 pandemic that began in Wuhan, China in December 2019 was declared a global emergency by the World Health Organization (WHO) in March 2020. As of October 7, 2021, there were almost 2 million confirmed COVID-19 cases with more than 27,000 deaths in Malaysia [1]. Given the high transmissibility of severe acute respiratory syndrome coronavirus 2 (SARS-CoV-2), the Ministry of Health (MOH) Malaysia has implemented strict guidelines to prevent and control COVID-19 infections among HCW. We, herein, report our valuable perioperative management in a paediatric patient undergoing emergency open appendicectomy during the COVID-19 pandemic. The patient's course was complicated by a false negative result for COVID-19 at initial testing, with a subsequent positive test result. We also highlight our challenges of using PPE and ways to address them. This work has been reported in line with the SCARE criteria [2].

## 2 Case presentation

An 11-year-old female (weight 29 kg, height 92 cm) presented to a district health clinic with a complaint of intermittent abdominal pain of one week duration, which was associated with low grade fever and episodes of loose stool. The patient described the pain as colicky and sharp in nature which was non radiating. She also had sore throat and non-productive dry cough for five days. However, she denied shortness of breath, anosmia, and close contact of confirmed COVID-19 patients. Their village house was located 20 km from the nearest township and had very difficult transportations due to the pandemic restrictions.

Our patient was initially treated as acute gastroenteritis (AGE) in a primary health care clinic and discharged with oral rehydration salt. However, her symptoms persisted and was referred to our tertiary centre. Prior to her transfer, an Antigen Rapid Test Kit (RTK-Ag) for COVID-19 was performed on her nasopharyngeal swab (NPS) specimen at the clinic, for which she tested negative.

On assessment at the emergency department (ED), the child was alert but appeared lethargic and dehydrated. She was febrile with a temperature of 37.8 °C. She was tachypnoeic with a respiratory rate (RR) of 25 breaths per minute and tachycardic with a heart rate (HR) of 125 beats per minute. Her blood pressure (BP) and oxygen saturation (SpO2) were 109/72 mmHg and 98% on room air, respectively. Further examination revealed a mildly distended abdomen with rebound tenderness over McBurney's point. There were crepitations auscultated at both lung bases. Airway assessment showed a Mallampati score of 1.

**Fig. 1:**
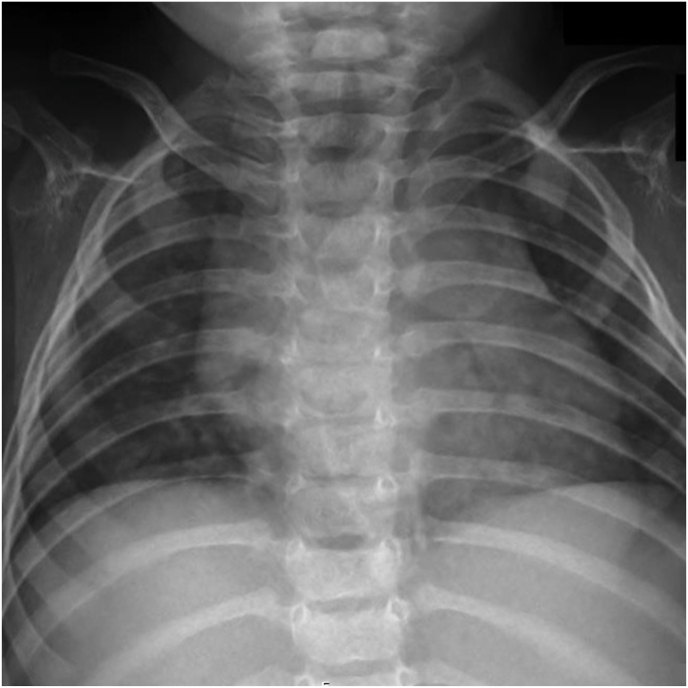
Chest X-ray of our patient showing ground glass appearances.

**Fig. 2:**
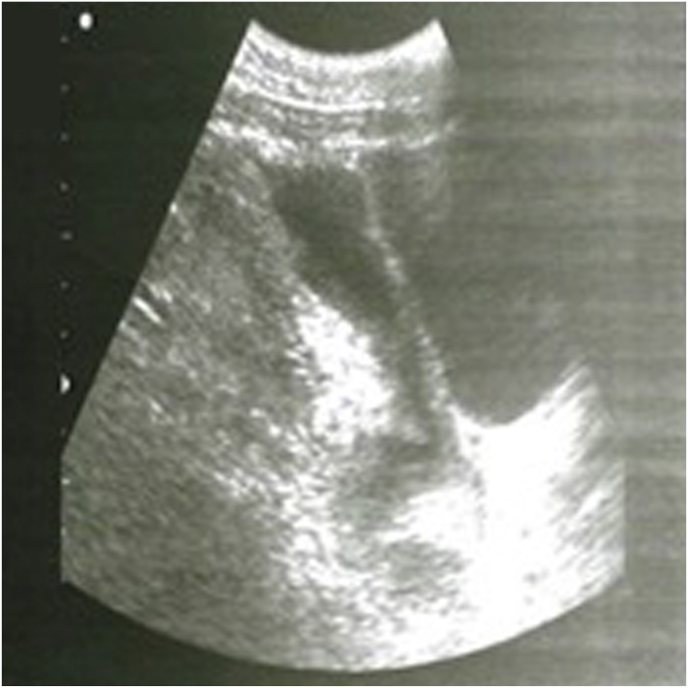
Bedside abdomen ultrasonography showing free fluids at the Pouch of Douglas.

Her NPS specimen was obtained and tested with COVID-19 real-time reverse transcription polymerase chain reaction (RT-PCR), which unfortunately was SARS-CoV-2 positive. She was immediately isolated. Her chest X-ray (CXR) showed bilateral ground glass opacifications (Fig. 1). Bedside ultrasonography of the abdomen showed free fluid in the pouch of Douglas (Fig. 2). The urine pregnancy test (UPT) was negative. Her full blood count revealed anaemia with a haemoglobin level of 110 g/L (Normal values: 115–135g/L). Both total white blood cell (TWBC) count and C-reactive protein (CRP) were raised at 20 × 10^9^/L (Normal values: 7–11 × 10^9^/L) and 128.8 mg/L (Normal values: < 3mg/L), respectively. All other biochemical results were within normal ranges.

Our immediate provisional diagnosis was perforated appendicitis with concurrent mild COVID-19 infection. Other differential diagnoses included ruptured tubo-ovarian abscess and pelvic inflammatory disease (PID).

She was posted for an emergency open appendicectomy after obtaining a high-risk consent from her parents. The patient was transferred to the designated operating room (OR) for COVID-19 patients. All involved HCW, namely, two general surgeons, anaesthetists, and nurses donned enhanced PPE including powered air-purifying respirators (PAPR) in the operating theatre (OT).

In the OR, standard American Society of Anesthesiologists (ASA) monitors were applied. Adequate preoxygenation was performed followed by modified rapid sequence induction (RSI) with administration of IV fentanyl 2 mcg/kg, propofol 3 mg/kg, and rocuronium 1 mg/kg with the application of cricoid pressure. Endotracheal intubation was facilitated using a C-MAC video laryngoscope. We applied two viral filters between the patient's endotracheal tube (ETT) and ventilator to minimize transmission. Synchronized intermittent mandatory ventilation volume-controlled ventilation (SIMV-VCV) mode were used to ventilate her lungs with fraction of inspired oxygen (FiO2) of 0.4, total oxygen and air mix flow of 1.8 L per minute, tidal volume of 6ml/kg and positive end expiratory pressure (PEEP) of 5cmH20, respectively. The patient's hemodynamics was stable and peak inspiratory pressure (PIP) generated ranged 19–22 cmH20.

The open appendicectomy was performed using a Lanz incision and her appendix was perforated, hence removed. The gynaecological structures appeared normal. The total blood loss was minimal and was replaced with adequate fluid therapy.

Postoperatively, the patient was extubated in the OR with IV sugammadex and subsequently transferred to a COVID-19 isolation ward. No growth of organisms was detected in her blood culture but *Escherichia coli* was isolated from the intraabdominal pus samples, which was sensitive to cefuroxime, trimethoprim-sulfamethoxazole, gentamicin, and amoxicillin-clavunalic acid. IV cefuroxime and metronidazole were administered and completed over 5 days postoperatively with no worsening sepsis. A repeated NPS RT-PCR 10 days later from her initial swab on admission tested negative for SARS-CoV-2 and she was discharged after a total of 14 days of admission.

## 3 Discussion

This case report is very significant in several aspects. First, there is a lack of published literatures on confirmed COVID-19 cases in the paediatric population globally. Second, it describes the importance of having high clinical suspicion when dealing with paediatric patients with upper respiratory tract infection (URTI). Third, it demonstrates the importance of PPE use among HCW to prevent and control exposure to COVID-19 infection as well as the issues associated with it. Fourth, it describes the measures taken to facilitate surgical procedures in COVID-19 patients in a resource-limited hospital.

See et al. highlighted the likelihood of false negatives from sampling error due to technical difficulty in obtaining NPS in paediatric patients [3]. This would explain the first negative RTK-Ag result turning positive on her subsequent RT-PCR test. A systematic review by Dinnes et al. concluded that when used in the first week after symptoms first developed, rapid antigen tests were most accurate [4]. Early studies from China reported that 65% of paediatric COVID-19 cases presented with features of URTI [5]. Ng et al. concluded that the presenting symptoms among the symptomatic COVID-19 cases mimicked common viral respiratory illnesses in childhood [6].

Certain measures had to be implemented to facilitate known or suspected COVID-19 cases needing surgery in a hospital, while at the same time safeguarding all HCW. First, security guards and attendants work together to clear the path between the origin and destination, to minimize risk of accidental contact during patient transfer. All HCW are required to wear enhanced PPE when handling COVID-19 patients. Biohazard signs are placed in the OT to restrict non-essential staff movements. This is because positive pressure generated in the OR to prevent entry of common pathogens can pose a risk of cross-contamination in the OR [7]. Contaminated routes and OR are immediately cleaned using sodium dichloroisocyanurate 2000 parts per million solutions for at least three times. Fourth, designated areas with guides displaying the correct sequence for donning and doffing PPE have been established in the OT. Errors such as lack of hand hygiene, improper disposal of PPE, and incorrect doffing sequence had been found to increase the risk of COVID-19 infection [8,9].

This case also reports the impact of COVID-19 on HCW. The use of PPE has detrimental impact on performance of manual tasks. Masks, respirators, face shields, goggles, and multi-layered gloves had impaired the HCW's visibility and dexterity. Anaesthetists struggled to palpate the child's veins due to multiple glove layering. They also spent more effort to operate the anaesthetic machine and monitors because the clear plastic drape affected the ergonomics of the appliances. Surgeons and nurses frequently encountered hampered awareness and verbal communication, causing them to speak louder and thus, additional discomfort. They were anxious due to concerns about the risk of being exposed to COVID-19 also lead to fear of committing medical errors.

COVID-19 had complicated routine medical care of our patient. To obtain informed consent for the surgery, a video conference was held between the surgeons and her parents. To make matters worse, the video calls kept disconnecting due to the poor internet connectivity at their rural village. In the OR, our patient was extremely uncooperative due to the HCW's near-faceless-astronaut appearance. She started crying uncontrollably to the extent that a video call with her parents was the only way she could calm her. Aggressive combatant paediatric patients requiring emergency surgery might necessitate the unnecessary use of anxiolytics and physical restraints [10].

Several steps were implemented to address our challenges in using PPE during the surgery. Each respective team of anaesthetists, surgeons, and nurses was led by a senior staff to provide good perioperative care. Besides that, to tackle the physical and mental distress, fully charged PAPR units were made available to every personnel in the OR. IV anaesthetic and emergency drugs were meticulously prepared based on her body weight so that medication errors could be avoided, before the anaesthetists don in the PAPR.

The nursing staffs consoled the child effectively prior to induction of anaesthesia. Needlestick injuries were avoided because by the time anaesthesia was induced, the patient had already calmed down. Sufficient fluids were judiciously addressed, avoiding fluid overload that would complicate her mild COVID-19 pneumonia. The ventilator settings were titrated to prevent barotrauma and low flow of gas mix to prevent aerosolization. The senior experienced surgeon was able to perform the procedure quickly, reducing operation time and minimising complications. Emergence from anaesthesia was smooth by giving sufficient analgesics and maintaining a comfortable environment.

Our experience with PPE and the problems associated, is consistent irrespective of the types of surgical procedures being done [[11], [12], [13], [14]]. The reports agreed that while PPE complicates resuscitation efforts and technical performance on manual tasks as well as significantly contributes to physical and mental exhaustion, it is necessary as it protects against HCW in this fight against the COVID-19 pandemic.

## 4 Conclusion

Healthcare providers must maintain a high index of suspicion of COVID-19 infection in children with URTI symptoms. Appropriate isolation, standard precautions, and PPE remain the first line of protection for HCW to prevent COVID-19 cross infection. Anaesthetists should be well trained in managing anxious children with COVID-19. HCW handling young children with COVID-19 must receive regular simulation and scenario-based training to be better prepared for pressurised situations.

## Patient's perspective

As a parent, I am very grateful to the anaesthetic and surgical teams for taking care of my daughter during the surgery. We were fortunate to have a kind, understanding and helpful teams for assisting us during this pandemic. We are glad that my daughter is safe and no cross infection among the healthcare workers.

## Ethical approval

This case report does not need any ethical approvals.

## Sources of funding

There are no funds received for this manuscript.

## Registration of research studies

Not related.

1. Name of the registry:

2. Unique Identifying number or registration ID:

3. Hyperlink to your specific registration (must be publicly accessible and will be checked):

## Author contribution

Dr Annie Maxwell and Dr Boon Tat Yeap were the clinicians involved in the management of the patient. They are the co-authors for this manuscript as well with Dr Kai Ming Teah and May Zaw Soe.

## Guarantor

BOON TAT YEAP.

## Consent

Informed and written consents were obtained from the patient and parents involved.

## Authors’ contribution

Dr Boon Tat Yeap and Dr Annie Maxwell were the clinicians and co-authors for this manuscript.

Dr Kai Ming Teah and Dr May Zaw Soe were co-authors for this manuscript and assisting in data collection.

## Registration of research studies

This is a case report. No human participants were involved.

## Guarantor

Dr Boon Tat Yeap is the guarantor for this manuscript.

## Consent

Written informed consent was obtained from the patient and parents for publication of this case report and any accompanying images. A copy of the written consent is available for review by the Editor-in-Chief of this journal.

## Provenance and peer review

Not commissioned, externally peer-reviewed.

## Declaration of competing interest

There is no conflict of interest in our manuscript.
